# 4-Iodo­anilinium perchlorate 18-crown-6 clathrate

**DOI:** 10.1107/S1600536811004260

**Published:** 2011-02-12

**Authors:** Yi Zhang, Min-Min Zhao

**Affiliations:** a Ordered Matter Science Research Center, Southeast University, Nanjing 211189, People’s Republic of China

## Abstract

In the title compound, C_6_H_7_IN^+^·ClO_4_^−^·C_12_H_24_O_6_, the proton­ated 4-iodo­anilinium cation inter­acts with the 18-crown-6 through three N—H⋯O hydrogen bonds, forming a rotator–stator-like structure. The cation, anion and 18-crown-6 mol­ecule all have crystallographically imposed mirror symmetry.

## Related literature

For the structure of a related 18-crown-6 clathrate, see: Ge & Zhao (2010 ▶). For ferroelectric properties, see: Fu *et al.* (2007 ▶); Ye *et al.*(2009 ▶); Zhang *et al.* (2009 ▶).
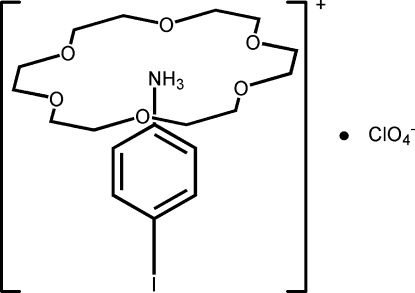


## Experimental

### 

#### Crystal data

C_6_H_7_IN^+^·ClO_4_^−^·C_12_H_24_O_6_
*M* *_r_* = 583.79
Orthorhombic, 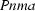

*a* = 15.8805 (11) Å
*b* = 11.3878 (11) Å
*c* = 12.6754 (8) Å
*V* = 2292.3 (3) Å^3^
*Z* = 4
Mo *K*α radiation
μ = 1.57 mm^−1^
*T* = 93 K
0.40 × 0.30 × 0.20 mm

#### Data collection

Rigaku SCXmini diffractometer
Absorption correction: multi-scan (*CrystalClear*; Rigaku, 2005 ▶) *T*_min_ = 0.575, *T*_max_ = 0.731
24048 measured reflections
2755 independent reflections
2611 reflections with *I* > 2σ(*I*)
*R*_int_ = 0.034

#### Refinement

*R*[*F* ^2^ > 2σ(*F* ^2^)] = 0.024
*wR*(*F* ^2^) = 0.093
*S* = 1.11
2755 reflections
154 parameters
H-atom parameters constrained
Δρ_max_ = 0.81 e Å^−3^
Δρ_min_ = −0.65 e Å^−3^

### 

Data collection: *CrystalClear* (Rigaku, 2005 ▶); cell refinement: *CrystalClear*; data reduction: *CrystalClear*; program(s) used to solve structure: *SHELXS97* (Sheldrick, 2008 ▶); program(s) used to refine structure: *SHELXL97* (Sheldrick, 2008 ▶); molecular graphics: *SHELXTL* (Sheldrick, 2008 ▶); software used to prepare material for publication: *SHELXTL*.

## Supplementary Material

Crystal structure: contains datablocks I, global. DOI: 10.1107/S1600536811004260/rz2549sup1.cif

Structure factors: contains datablocks I. DOI: 10.1107/S1600536811004260/rz2549Isup2.hkl

Additional supplementary materials:  crystallographic information; 3D view; checkCIF report

## Figures and Tables

**Table 1:** Hydrogen-bond geometry (Å, °)

*D*—H⋯*A*	*D*—H	H⋯*A*	*D*⋯*A*	*D*—H⋯*A*
N2—H2*A*⋯O4^i^	0.90	1.96	2.861 (2)	176
N2—H2*C*⋯O2^i^	0.90	1.95	2.854 (3)	178

Symmetry code: (i) 
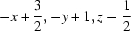
.

**Fig. 1.**
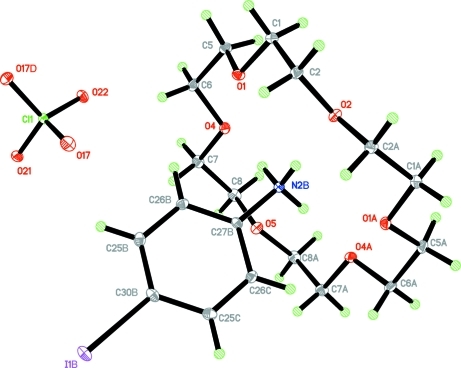
The molecular structure of the title compound, with the atomic numbering scheme. Displacement ellipsoids are drawn at the 30% probability level. Symmetry codes: (A) x, 1/2-y, z; (B) 3/2-x, 1-y, 1/2+z; (C) 3/2-x, -1/2+y, 1/2+z; (D) x, 3/2-y, z.

**Fig. 2.**
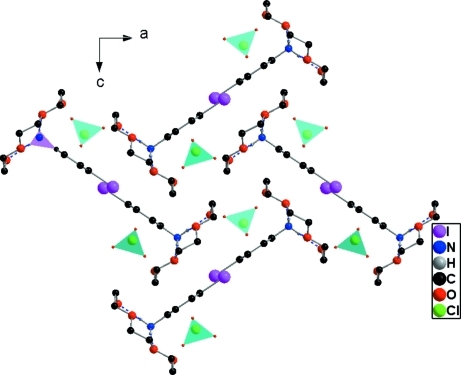
Packing diagram of the title compound viewed along the b axis. Dashed lines indicate hydrogen bonds. Hydrogen atoms not involved in hydrogen bonding are omitted for clarity.

## supplementary crystallographic information

### Comment

As a continuation of our studies on the synthesis and characterization of host-guest compounds of 18-crown-6 (Ge & Zhao, 2010), the crystal structure of the title compound is reported herein. The dielectric permittivity of the title compound was tested to investigate the ferroelectric phase transitions materials (Fu *et al.* 2007; Ye *et al.* 2009; Zhang *et al.* 2009). The title compound have no dielectric anomalies under 1*M* Hz in the temperature range from 90 to 473 K (the compound m. p. > 473 K), suggesting that in the compound no distinct phase transition occurred within the measured temperature range.

The title compound is composed of C_6_H_4_INH_3_^+^ cations, 18-crown-6 molecules and ClO_4_^-^ anions (Fig 1). A supramolecular rotator-stator structure is assembled between the protonated 4-iodoanilinium cation and 18-crown-6 molecule by three N—H···O hydrogen bonds (Table 1). The nitrogen atom of the NH_3_^+^ group is in the perching position of the crown ring, rather than in the nesting position. The macrocycle adopts a conformation with approximate D_3d_ symmetry, all O—C—C—O torsion angles being *gauche* and alternating in sign, and all C—O—C—C torsion angles being *trans*. The C—N bond of the 4-iodoanilinium cation is almost perpendicular to the mean plane of the crown-ether O atoms. The ClO_4_^-^ anion, the cation and the 18-crown-6 have all crystallographically imposed mirror symmetry. Fig. 2 shows a view of the structure down the *b* axis. The couples of head-to-head rotator-stator cations almost paralleling and plumbing the (101) direction are alternating arranged. The anions inhabit the cavities formed by the couples of head-to-head rotator-stator cations. In the title compound no intermolecular hydrogen bonds are observed.

### Experimental

4-Iodoaniline (2 mmol) and excess perchloric acid (4 mmol) were dissolved in methanol, then 18-crown-6 (2 mmol) was added to the mixture. The precipitate was filtered and washed with a small amount of methanol. Single crystals suitable for X-ray diffraction analysis were obtained from slow evaporation of the methanol solution at room temperature after two days.

### Refinement

All hydrogen atoms were calculated geometrically allowed to ride, with C—H = 0.93-0.97 Å, N—H = 0.90 Å, and with *U*_iso_(H) = 1.2 *U*_iso_(C, N).

### Crystal data

**Table:**

C_6_H_7_IN^+^·ClO_4_^−^·C_12_H_24_O_6_	*F*(000) = 1184
*M**_r_* = 583.79	*D*_x_ = 1.692 Mg m^−^^3^
Orthorhombic, *P**n**m**a*	Mo *K*α radiation, λ = 0.71073 Å
Hall symbol: -P 2ac 2n	Cell parameters from 17071 reflections
*a* = 15.8805 (11) Å	θ = 3.2–27.8°
*b* = 11.3878 (11) Å	µ = 1.57 mm^−^^1^
*c* = 12.6754 (8) Å	*T* = 93 K
*V* = 2292.3 (3) Å^3^	Prism, colourless
*Z* = 4	0.40 × 0.30 × 0.20 mm

### Data collection

**Table:**

Rigaku SCXmini diffractometer	2755 independent reflections
Radiation source: fine-focus sealed tube	2611 reflections with *I* > 2σ(*I*)
graphite	*R*_int_ = 0.034
Detector resolution: 13.6612 pixels mm^-1^	θ_max_ = 27.5°, θ_min_ = 2.1°
CCD_Profile_fitting scans	*h* = −20→20
Absorption correction: multi-scan (*CrystalClear*; Rigaku, 2005)	*k* = −14→14
*T*_min_ = 0.575, *T*_max_ = 0.731	*l* = −16→16
24048 measured reflections	

### Refinement

**Table:**

Refinement on *F*^2^	Primary atom site location: structure-invariant direct methods
Least-squares matrix: full	Secondary atom site location: difference Fourier map
*R*[*F*^2^ > 2σ(*F*^2^)] = 0.024	Hydrogen site location: inferred from neighbouring sites
*wR*(*F*^2^) = 0.093	H-atom parameters constrained
*S* = 1.11	*w* = 1/[σ^2^(*F*_o_^2^) + (0.0591*P*)^2^ + 1.2714*P*] where *P* = (*F*_o_^2^ + 2*F*_c_^2^)/3
2755 reflections	(Δ/σ)_max_ < 0.001
154 parameters	Δρ_max_ = 0.81 e Å^−^^3^
0 restraints	Δρ_min_ = −0.65 e Å^−^^3^

### Special details

**Table:**

Geometry. All e.s.d.'s (except the e.s.d. in the dihedral angle between two l.s. planes) are estimated using the full covariance matrix. The cell e.s.d.'s are taken into account individually in the estimation of e.s.d.'s in distances, angles and torsion angles; correlations between e.s.d.'s in cell parameters are only used when they are defined by crystal symmetry. An approximate (isotropic) treatment of cell e.s.d.'s is used for estimating e.s.d.'s involving l.s. planes.
Refinement. Refinement of *F*^2^ against ALL reflections. The weighted *R*-factor *wR* and goodness of fit *S* are based on *F*^2^, conventional *R*-factors *R* are based on *F*, with *F* set to zero for negative *F*^2^. The threshold expression of *F*^2^ > σ(*F*^2^) is used only for calculating *R*-factors(gt) *etc*. and is not relevant to the choice of reflections for refinement. *R*-factors based on *F*^2^ are statistically about twice as large as those based on *F*, and *R*- factors based on ALL data will be even larger.

### Fractional atomic coordinates and isotropic or equivalent isotropic displacement parameters (Å^2^)

**Table:**

	*x*	*y*	*z*	*U*_iso_*/*U*_eq_	
I1	1.020717 (13)	0.7500	0.497027 (14)	0.01781 (10)	
N2	0.69020 (16)	0.7500	0.21864 (19)	0.0118 (5)	
H2A	0.6919	0.6850	0.1782	0.018*	
H2C	0.6440	0.7500	0.2599	0.018*	
C25	0.87428 (14)	0.6439 (2)	0.37576 (16)	0.0155 (4)	
H25A	0.8994	0.5711	0.3975	0.019*	
C26	0.80150 (13)	0.64394 (18)	0.31502 (16)	0.0141 (4)	
H26A	0.7757	0.5713	0.2941	0.017*	
C27	0.76611 (18)	0.7500	0.2846 (2)	0.0116 (5)	
C30	0.91063 (18)	0.7500	0.4050 (2)	0.0146 (6)	
O4	0.79657 (9)	0.45595 (13)	0.59043 (12)	0.0152 (3)	
O5	0.70915 (15)	0.2500	0.52965 (18)	0.0158 (4)	
C6	0.81605 (14)	0.56511 (19)	0.64275 (17)	0.0160 (4)	
H6A	0.7730	0.5830	0.6947	0.019*	
H6B	0.8177	0.6286	0.5917	0.019*	
C7	0.71692 (14)	0.4594 (2)	0.53731 (19)	0.0176 (5)	
H7A	0.7124	0.5309	0.4961	0.021*	
H7B	0.6715	0.4584	0.5885	0.021*	
C8	0.71071 (15)	0.3540 (2)	0.46600 (18)	0.0182 (5)	
H8A	0.6598	0.3587	0.4239	0.022*	
H8B	0.7586	0.3518	0.4186	0.022*	
O1	0.89363 (9)	0.46966 (13)	0.78008 (12)	0.0141 (3)	
O2	0.95815 (15)	0.2500	0.84574 (18)	0.0137 (4)	
C1	0.97204 (13)	0.4574 (2)	0.83409 (18)	0.0155 (4)	
H1A	0.9841	0.5282	0.8739	0.019*	
H1B	1.0171	0.4456	0.7835	0.019*	
C2	0.96711 (14)	0.35433 (19)	0.90738 (17)	0.0150 (4)	
H2D	1.0178	0.3498	0.9499	0.018*	
H2E	0.9193	0.3629	0.9543	0.018*	
Cl1	0.60734 (4)	0.7500	0.70231 (5)	0.01306 (16)	
O17	0.62206 (10)	0.64643 (15)	0.76547 (13)	0.0230 (4)	
O21	0.52124 (13)	0.7500	0.6644 (2)	0.0173 (5)	
O22	0.66395 (14)	0.7500	0.61313 (17)	0.0179 (5)	
C5	0.90019 (14)	0.55317 (19)	0.69569 (17)	0.0157 (4)	
H5A	0.9419	0.5269	0.6450	0.019*	
H5B	0.9179	0.6287	0.7233	0.019*	

### Atomic displacement parameters (Å^2^)

**Table:**

	*U*^11^	*U*^22^	*U*^33^	*U*^12^	*U*^13^	*U*^23^
I1	0.01152 (15)	0.02558 (16)	0.01632 (15)	0.000	−0.00231 (6)	0.000
N2	0.0115 (11)	0.0107 (11)	0.0132 (11)	0.000	0.0000 (10)	0.000
C25	0.0162 (10)	0.0170 (10)	0.0135 (9)	0.0027 (8)	0.0001 (8)	0.0020 (8)
C26	0.0154 (10)	0.0123 (10)	0.0145 (10)	−0.0008 (8)	0.0009 (8)	−0.0015 (8)
C27	0.0095 (13)	0.0148 (14)	0.0104 (13)	0.000	0.0009 (11)	0.000
C30	0.0101 (13)	0.0228 (15)	0.0108 (13)	0.000	0.0019 (11)	0.000
O4	0.0136 (7)	0.0131 (7)	0.0189 (7)	0.0020 (6)	−0.0022 (6)	0.0007 (6)
O5	0.0195 (11)	0.0148 (11)	0.0131 (10)	0.000	−0.0012 (9)	0.000
C6	0.0195 (10)	0.0107 (10)	0.0177 (10)	0.0017 (8)	0.0015 (9)	0.0025 (8)
C7	0.0152 (10)	0.0195 (11)	0.0180 (11)	0.0027 (8)	−0.0034 (9)	0.0037 (9)
C8	0.0181 (11)	0.0235 (12)	0.0128 (10)	0.0002 (9)	−0.0026 (9)	0.0047 (9)
O1	0.0130 (7)	0.0137 (7)	0.0156 (7)	−0.0019 (5)	−0.0003 (6)	0.0031 (6)
O2	0.0184 (10)	0.0099 (10)	0.0128 (10)	0.000	−0.0013 (9)	0.000
C1	0.0137 (10)	0.0138 (11)	0.0189 (11)	−0.0020 (8)	−0.0009 (8)	−0.0013 (8)
C2	0.0158 (10)	0.0142 (10)	0.0151 (10)	0.0002 (8)	−0.0015 (8)	−0.0027 (8)
Cl1	0.0118 (3)	0.0126 (3)	0.0148 (3)	0.000	0.0005 (3)	0.000
O17	0.0210 (8)	0.0224 (9)	0.0257 (9)	0.0028 (7)	0.0004 (7)	0.0111 (7)
O21	0.0116 (11)	0.0177 (12)	0.0225 (13)	0.000	−0.0016 (8)	0.000
O22	0.0154 (11)	0.0215 (11)	0.0169 (11)	0.000	0.0053 (9)	0.000
C5	0.0188 (10)	0.0120 (10)	0.0163 (10)	−0.0030 (8)	0.0027 (8)	0.0021 (8)

### Geometric parameters (Å, °)

**Table:**

I1—C30	2.102 (3)	C7—H7A	0.9700
N2—C27	1.467 (4)	C7—H7B	0.9702
N2—H2A	0.9002	C8—H8A	0.9700
N2—H2C	0.9006	C8—H8B	0.9700
C25—C26	1.389 (3)	O1—C1	1.428 (3)
C25—C30	1.390 (3)	O1—C5	1.435 (2)
C25—H25A	0.9599	O2—C2	1.429 (2)
C26—C27	1.387 (3)	O2—C2^ii^	1.429 (2)
C26—H26A	0.9601	C1—C2	1.499 (3)
C27—C26^i^	1.387 (3)	C1—H1A	0.9699
C30—C25^i^	1.390 (3)	C1—H1B	0.9700
O4—C7	1.433 (3)	C2—H2D	0.9701
O4—C6	1.443 (3)	C2—H2E	0.9701
O5—C8^ii^	1.433 (3)	Cl1—O22	1.444 (2)
O5—C8	1.433 (3)	Cl1—O17^i^	1.4445 (16)
C6—C5	1.501 (3)	Cl1—O17	1.4445 (16)
C6—H6A	0.9700	Cl1—O21	1.449 (2)
C6—H6B	0.9701	C5—H5A	0.9701
C7—C8	1.506 (3)	C5—H5B	0.9700
			
C27—N2—H2A	107.4	C7—C8—H8A	110.0
C27—N2—H2C	109.7	O5—C8—H8B	109.9
H2A—N2—H2C	110.8	C7—C8—H8B	109.9
C26—C25—C30	119.5 (2)	H8A—C8—H8B	108.3
C26—C25—H25A	120.4	C1—O1—C5	111.03 (16)
C30—C25—H25A	120.1	C2—O2—C2^ii^	112.5 (2)
C27—C26—C25	119.5 (2)	O1—C1—C2	109.15 (17)
C27—C26—H26A	120.0	O1—C1—H1A	109.9
C25—C26—H26A	120.5	C2—C1—H1A	109.8
C26^i^—C27—C26	121.1 (3)	O1—C1—H1B	109.9
C26^i^—C27—N2	119.44 (13)	C2—C1—H1B	109.8
C26—C27—N2	119.44 (14)	H1A—C1—H1B	108.3
C25—C30—C25^i^	120.9 (3)	O2—C2—C1	108.51 (18)
C25—C30—I1	119.56 (14)	O2—C2—H2D	110.0
C25^i^—C30—I1	119.56 (14)	C1—C2—H2D	110.0
C7—O4—C6	112.42 (16)	O2—C2—H2E	110.0
C8^ii^—O5—C8	111.4 (2)	C1—C2—H2E	110.0
O4—C6—C5	108.56 (17)	H2D—C2—H2E	108.4
O4—C6—H6A	110.0	O22—Cl1—O17^i^	109.46 (9)
C5—C6—H6A	110.0	O22—Cl1—O17	109.46 (9)
O4—C6—H6B	110.0	O17^i^—Cl1—O17	109.47 (15)
C5—C6—H6B	110.0	O22—Cl1—O21	109.14 (14)
H6A—C6—H6B	108.4	O17^i^—Cl1—O21	109.65 (9)
O4—C7—C8	108.52 (18)	O17—Cl1—O21	109.65 (9)
O4—C7—H7A	110.0	O1—C5—C6	109.19 (17)
C8—C7—H7A	109.9	O1—C5—H5A	109.8
O4—C7—H7B	110.0	C6—C5—H5A	109.9
C8—C7—H7B	110.0	O1—C5—H5B	109.9
H7A—C7—H7B	108.4	C6—C5—H5B	109.8
O5—C8—C7	108.78 (18)	H5A—C5—H5B	108.3
O5—C8—H8A	109.9		

Symmetry codes: (i) *x*, −*y*+3/2, *z*; (ii) *x*, −*y*+1/2, *z*.

### Hydrogen-bond geometry (Å, °)

**Table:**

*D*—H···*A*	*D*—H	H···*A*	*D*···*A*	*D*—H···*A*
N2—H2A···O4^iii^	0.90	1.96	2.861 (2)	176
N2—H2C···O2^iii^	0.90	1.95	2.854 (3)	178

Symmetry codes: (iii) −*x*+3/2, −*y*+1, *z*−1/2.

## References

[bb1] Fu, D. W., Song, Y. M., Wang, G. X., Ye, Q., Xiong, R. G., Akutagawa, T., Nakamura, T., Chan, P. W. H. & Huang, S. P. (2007). *J. Am. Chem. Soc.* **129**, 5346–5347. 10.1021/ja0701816 17428055

[bb2] Ge, J.-Z. & Zhao, M.-M. (2010). *Acta Cryst.* E**66**, o1478. 10.1107/S1600536810019033 PMC2979430 21579544

[bb3] Rigaku (2005). *CrystalClear* Rigaku Corporation, Tokyo, Japan.

[bb4] Sheldrick, G. M. (2008). *Acta Cryst.* A**64**, 112–122. 10.1107/S0108767307043930 18156677

[bb5] Ye, H. Y., Fu, D. W., Zhang, Y., Zhang, W., Xiong, R. G. & Huang, S. P. (2009). *J. Am. Chem. Soc.* **131**, 42–43. 10.1021/ja808331g 19128170

[bb6] Zhang, W., Cheng, L. Z., Xiong, R. G., Nakamura, T. & Huang, S. P. (2009). *J. Am. Chem. Soc.* **131**, 12544–12545. 10.1021/ja905399x 19685869

